# How to Save a Drowning Person

**Published:** 1882-05

**Authors:** 


**How to Save a Drowning Person.**—It may not be generally known that when a person is drowning, if he is taken by the arm from behind, between the elbow and shoulder, he can not touch the person attempting to save him, and whatever struggles he may make will only assist the person holding him in keeping his head above water. A good swimmer can keep a man thus above water for an hour. If seized any where else the probability is that he will clutch the swimmer, and perhaps, as is often the case, both will be drowned.

### COOKERY.

THE first, the indispensable requisites of healthful cooking are that the materials should be fresh, perfect, and ripe, and that they should be properly cooked. It may be safe to say that half of the food prepared for American tables is ruined, for all purposes of healthful nutrition, in the cooking, and destroys rather than builds up; weakens, instead of imparting vigor, and engenders wasting disease, rather than promotes good health. The higher classes of society, the best informed, appreciate these truths and seek to secure all their advantages by a more strict personal attention to the larder and the kitchen than those below them in the social scale; hence Professor Blot's observation that wherever he has gone to give practical scientific lectures on cooking, nine-tenths of his patrons have been among the elite of society; the wealthiest and the best informed, and this, too, when the price of admission has been so low as not to make it burdensome to the shortest, shallowest purse; and yet it is more important to the poor

